# “The History of Chinese Medicine Really Is Very Detailed Regarding Pandemics”: A Qualitative Analysis of Evidence-Based Practice and the Use of Chinese Herbal Medicine by Licensed Acupuncturists During the COVID-19 Pandemic in the United States

**DOI:** 10.1089/jicm.2023.0033

**Published:** 2023-11-09

**Authors:** Belinda J. Anderson, Melissa Zappa, Barbara Glickstein, Lisa Taylor-Swanson

**Affiliations:** ^1^ College of Health Professions, Pace University, New York, NY, USA.; ^2^ Albert Einstein College of Medicine, Bronx, NY, USA.; ^3^ Huntsman Cancer Institute, University of Utah, Salt Lake City, UT, USA.; ^4^ Barbara Glickstein Strategies, New York, NY, USA.; ^5^ College of Nursing, University of Utah, Salt Lake City, UT, USA.

**Keywords:** Chinese herbal medicine, COVID-19, pandemic, evidence-based medicine

## Abstract

**Objective:**

The objective of this qualitative study was to understand how licensed acupuncturists determined treatment strategies for patients with symptoms likely related to COVID-19 using Chinese herbal medicine (CHM) and the impact of the pandemic upon their clinical practice.

**Methods:**

A qualitative instrument was developed with questions aligned with when participants started treating patients with symptoms likely related to COVID-19 and the availability of information related to the use of CHM for COVID-19. Interviews took place between March 8 and May 28, 2021, and were transcribed verbatim by a professional transcription service. Inductive theme analysis and ATLAS.ti Web software were used to determine themes.

**Results:**

Theme saturation was achieved after 14 interviews lasting 11–42 min. Treatment predominantly started before mid-March 2020. Four themes emerged (1) information sources; (2) diagnostic and treatment decision-making; (3) practitioner experience; (4) resources and supplies.

**Conclusion:**

Primary sources of information informing treatment strategies came from China through professional networks and were widely disseminated throughout the United States. Scientific studies evaluating the effectiveness of CHM for COVID-19 were generally not deemed useful for informing patient care because treatment had been initiated before they were published and because of limitations associated with the research and the ability to apply it to real world practice.

## Introduction

In January 2020 the first COVID-19 infected patient was identified in the United States in Washington State after returning from Wuhan, China. In March 2020, states throughout the United States began to implement shutdowns to prevent the spread of the severe acute respiratory syndrome (SARS)-CoV-2 virus. At that time there was no biomedical cure or treatment for COVID-19 infection. In October 2020 when the U.S. Food and Drug Administration approved the first antiviral drug (remdesivir) for treating COVID-19 infection, ∼215,000 people had died from COVID-19 infection in the United States and over one million globally.^1^ The use of vaccines against COVID-19 was initiated in the United States in December 2020. Despite treatments and vaccines, the total number of deaths from COVID-19 infection in the United States in 2021 exceeded that in 2020.^2^

In China treatment for COVID-19 infection used Traditional Chinese Medicine (TCM) alone and in combination with biomedical treatment.^3,4^ TCM is a modernized form and practice style of the broader discipline of East Asian medicine (EAM) and was part of the official Chinese government prevention and control strategies for COVID-19.^5^ Chinese herbal medicine (CHM) was an important part of the Chinese government prevention and control strategies for COVID-19 probably because of the known antiviral properties of certain Chinese herbs,^6^ and the fact that CHM had been used extensively in prior epidemics.^7,8^ The use of CHM for epidemics similar to the COVID-19 pandemic throughout the long history of EAM was a significant factor that shaped the development of EAM theories and treatment approaches for all diseases.^6,9^

In EAM patients are diagnosed within the context of EAM theory, which then leads to the determination of a treatment principle and the selection of approaches, which can include acupuncture, CHM, moxibustion, cupping, and various other modalities. Patient treatment is individualized, and there is an emphasis on basing treatment upon the patient's presenting objective and subjective signs and symptoms. This is often referred to as “treat what you see.” Tongue and pulse analysis are important objective diagnostic techniques. CHM prescriptions (called formulas) are specific to individual patients and typically consist of 10–15 different constituents selected from the CHM pharmacopeia that contains over 500 different constituents. Practitioners usually start with a commonly used formula and then modify for individual patients by adding and/or removing specific constituents. This is termed formula modification.

Research examining the efficacy and effectiveness of CHM against COVID-19 developed rapidly. Initially, in the first half of 2020 published research studies were mainly observational (case studies and case series).^4^ From mid 2020 onward, randomized controlled trials were published, and more recently, many systematic reviews and meta-analyses of these trials have been published.^10–13^ These studies conclude that there is encouraging evidence to support the effectiveness and clinical usage of CHM for COVID-19 symptoms and recommend further research. The randomized controlled trials included in the systematic reviews had a low to medium risk of bias.

The mechanistic basis for the effectiveness of CHM against the SARS-CoV-2 virus has also received intense research focus with several mechanistic pathways being discovered.^14,15^ These lines of investigation examine the antiviral properties of CHM. Although such research provides a possible understanding of the way in which CHM may be effective for COVID-19, it is often not the basis upon which clinical practice is based. CHM is usually prescribed within the theoretical diagnostic constructs of EAM and often involves the use of ancient formulas that are modified for individual patients. As such, the antiviral properties of certain herbs may not be a significant factor in the selection of herbs for patient treatment.

Information about the use of CHM in China to treat COVID-19 infection rapidly spread throughout the world. This led to the therapeutic use of CHM to treat COVID-19 in many other countries, including the United States by licensed EAM practitioners (usually referred to as licensed acupuncturists). The lack of biomedical treatments for COVID-19, along with vaccine hesitancy and ongoing high COVID-19 associated death rates, created compelling circumstances for the continued demand for treatment approaches that had evidence of effectiveness from prior recent epidemics.^7,16^

In April 2021 the authors initiated a mixed-methods study of U.S. licensed acupuncturists who had treated five or more patients with CHM for symptoms likely related to COVID-19.^17^ The focus of their study was evidence-based practice. Their aim was to investigate how licensed acupuncturists decided upon treatment strategies and how the pandemic impacted their practice. The quantitative part is published elsewhere^17^ and consisted of a 28-question survey. Their findings demonstrated that acupuncturists used a wide variety of information sources in devising treatment approaches. These included information from China about the use of CHM for COVID-19 that was disseminated through collegial networks, continuing education providers, CHM companies, and EAM journals. Other important information sources were biomedical journals, use of CHM for past viral outbreaks and epidemics, and their own clinical experience.

This article reports on the qualitative aspect of their study with the aim being to further understand the treatment making decision process and the impact of the COVID-19 pandemic upon practitioner's clinical practice.

## Methods

### Qualitative instrument

A structured questionnaire instrument was prepared by the research team. It was designed to elucidate the use of information and evidence by licensed acupuncturists prescribing CHM in their decision-making process of treating patients with symptoms likely related to COVID-19. All interviews started with the interviewer asking for informed consent, indicating that a series of questions would be asked, stating that there were no right or wrong answers and acknowledging respect for clinicians devising treatment strategies in many ways. The latter was included to reduce the possibility of bias related to participants feeling that they should have been aware of possible evidence sources and used them in clinical practice.

Participants were first asked when they started treating patients that had symptoms that may be related to COVID-19. The authors divided the early stages of the COVID-19 pandemic into three phases as defined by the availability of information about the use of CHM for COVID-19. Phase I was early/mid-March 2020, which was when there appeared to be little to no specific information about the use or effectiveness of CHM for COVID-19. Phase II was late March/April 2020 when anecdotal information and reports of effectiveness of CHM for COVID patients based on their use in China began to be disseminated in the United States. Phase III was June 2020 onward when scientific studies and later systematic reviews exploring the effectiveness of CHM for COVID-19 were available in biomedical journals.

Having established in which phase the practitioner had started treating patients with symptoms likely related to COVID-19, questions that followed were tailored around this response. Participants who started in Phase I were asked how they knew which CHM formulas to use assuming they did not have access to information specific to the use of CHM for COVID-19 to guide their decisions. Phase I participants were then asked the same questions as those who started in Phases II and III. Participants who started in Phases II and III were asked if they knew about the anecdotal information and later scientific studies about the use of CHM for COVID-19 that was disseminated to the United States. If they answered yes, they were then asked how it impacted their treatment approach. If they answered no, they were asked how they knew which CHM formulas to use for their patients. All participants were also asked about patient access to COVID-19 testing and encouraged to share additional information.

### Participant recruitment

Survey participants were recruited by disseminating invitations among the author's colleagues, paid advertisements through Acupuncture Today, and by creating a “teamcovidstudy” website (now defunct). Eligible participants had to be licensed acupuncturists in the United States who had treated more than five patients with symptoms likely related to COVID-19. The quantitative survey portion of this study^17^ also included an invitation to be interviewed. Interest was expressed by e-mailing the principal investigator (B.J.A.) who then connected them to the interviewer (B.G.). A convenience sampling method was used whereby participants were contacted to set up an interview in the chronological order that the requests for participation were received. Further recruitment and interviews were terminated when qualitative theme saturation was attained. No compensation was provided to participants. Participants were given participant codes (PC) for the purposes of identifying their contributions in this publication.

### Interview implementation

This qualitative study was approved by the Institutional Review Board of Albert Einstein College of Medicine (Einstein, IRB No. 2020-12556). Informed consent was provided verbally to participants at the beginning of the interviews. Interviews were conducted by B.G. who has extensive experience with qualitative and investigative interviewing and has no direct ties or clinical experience with CHM. During the project design phase B.G. was involved in discussions about the use of CHM for COVID, the historical use of CHM for prior epidemics, and provided access to websites and publications outlining the use of CHM for COVID-19. Interviews were conducted between March 8, 2021, and May 28, 2021. All interviews were transcribed verbatim by a professional transcription service. To maintain confidentiality, only the deidentified audio recordings and transcription documents of each interview were shared with the other investigators (B.J.A., M.Z., and L.T.-S.). M.Z. listened to each recording and compared with the transcribed documents to ensure accuracy and clarification of any Chinese medical or herbal terminology.

### Data analysis

Data analysis was undertaken by B.J.A., M.Z., and L.T.-S. using predominantly an inductive content analysis methodology^18^ and using ATLAS.ti Web (Version 22.1) software.^19^ Outcomes from the survey component of the project were also taken into consideration in determining themes. Data analysis was initiated, while interviews were being conducted to enable determination of saturation as defined by Guest et al.^20^—“the point in data collection and analysis when new information produces little or no change to the codebook.” Transcripts were analyzed independently by B.J.A., M.Z., and L.T.-S. to first identify codes. B.J.A., M.Z., and L.T.-S. then met to refine the codes and then combine these into themes and subthemes. Regular meetings facilitated consensus building. M.Z. collated summaries of agreed upon codes and themes to track data analysis progress and created master lists of emerging codes and themes.

The data were analyzed through a pragmatic worldview inclusive of an embedded secondary mixed methods strategy^21^ in which codes were counted and examined quantitatively as part of the subsequent theme analysis.^22–24^ Quantitative analysis of the data was undertaken by uploading the transcribed interviews into the ATLAS.ti software and using the master list of 51 individual codes. If excerpts of participant responses contained two or more codes, all applicable codes were applied to that same selection to preserve depth of information. Quantitative analysis permitted verification of the relative importance of the different themes and subthemes. Geographical location of participants and when they started treating COVID-19 patients with CHM (phase I, II, or III) were coded and categorized separately.

B.J.A., L.T.-S., and M.Z. are licensed acupuncturists and CHM practitioners. B.J.A. and M.Z. are certified in CHM by the National Certification Commission for Acupuncture and Oriental Medicine (NCCAOM).^25^ The authors used the Standards for Reporting Qualitative Research (SRQR) in writing their report.^26^

## Results

Twenty-eight people requested to be interviewed. Theme saturation was achieved after interviewing 14 participants. Interviews lasted between 11 and 42 min. Participants represented eight different states in the United States with a predominance in California (Table 1).

**Table 1.** Location of Participants and Timing of Patient Treatment

Location of participant	
State	No. of participants
California	6
New York	2
Colorado	1
Illinois	1
New Jersey	1
North Carolina	1
Texas	1
Vermont	1
	14

*Timing*	*No.* ^a^
Phase 1—early/mid-March 2020	10
Phase II—late March/April 2020	3
Before early/mid-March	9

^a^ Total number of times recorded across all interviews.

### Phase

Most participants reported treating patients with symptoms probably related to COVID-19 in the very early stages of the pandemic (Table 1) with several thinking they had seen patients before COVID-19 being officially recognized in the U.S.—“I didn't know it at the time, but I probably saw someone in late February” (PC 6), “I actually believe we had some in November and December of 2019. It was a very unusual cold and flu season where I actually quarantined off one specific room only for cold and flu, so I believe it was before then, looking back” (PC 13).

### Themes

Four themes emerged from participant interviews (Tables 2–5)—(1) information sources; (2) diagnostic and treatment decision-making; (3) practitioner experience; (4) resources and supplies.

**Table 2.** Information Sources

Theme	Subtheme	No.*^a^*
Anecdotal sources	Indirectly from China through colleagues	9
	John Chen	7
	CHM formulas used in China	7
	Direct contact with colleagues in China	6
	E Lotus	2
	The Lantern	1
	Heiner Fruehauf	1
Scientific studies		
Awareness/usage	Aware	16
	Aware but did not use	10
	Not aware	2
	Used	7
Limitations to use		
	Not reflective of real-world cases	4
	Seeing CHM through biomedical framework	3
	Information from China not suitable for early or mild COVID-19	2
	Patients in U.S. are different or at a different COVID stage	2

^a^ Total number of times the theme was mentioned across all interviews.

CHM, Chinese herbal medicine.

**Table 3.** Diagnostic and Treatment Decision-Making

Theme	Subtheme	No.*^a^*
Flexible thinking and adaptation	“Treat what I see”	16
	Use of first principles	12
	Adapted for local conditions	1
	Created own evidence pyramid	1
Clinical experience	Confident of own clinical skills^b^	12
	Success with own formulas	5
Treatments influenced by	Senior mentors	6
	Local U.S. colleagues^b^	10
	Colleagues in China^c^	6
	Articles	11^d^
	COVID test status	12^e^
	Use of CHM in prior epidemics	2

^a^ Total number of times the theme was mentioned across all interviews.

^b^ Overlap with Table 3—practitioner experience.

^c^ Overlap with Table 1—Anecdotal sources.

^d^ Seven were from biomedical journals.

^e^ Ten participants reported that a positive COVID test did not influence their treatment, and two said that a positive test increased the seriousness of the case.

CHM, Chinese herbal medicine.

**Table 4.** Practitioner Experience

Theme	Subtheme	No.*^a^*
Confident of own clinical skills^b^		12
Collaboration	Other EAM practitioners	10
	Quick coming together of the global EAM community	4
Telehealth	Used telehealth	8
	Limitations of telehealth^c^	3
Treatment success	Success with long-COVID	7
	Kept patients out of hospital	1
Treated mainly COVID positive patients		4
Delivered CHM to patient's home		4
Prescribed CHM for prevention		3
Used knowledge from EAM and biomedicine		2

^a^ Total number of times the theme was mentioned across all interviews.

^b^ Overlap with Table 2—Treatments influenced by.

^c^ Overlap with Table 4—Telehealth.

CHM, Chinese herbal medicine; EAM, East Asian medicine.

**Table 5.** Barriers and Limitations Associated with Resources and Supplies

Theme	Subtheme	No.*^a^*
CHM	Supply chain restrictions	10
	Had to substitute different herbs	5
	Stocked up personal supply	7
COVID testing	Patients unable to obtain	10
	Patients able to obtain	5
Disruption to CHM patient delivery services		4
Telehealth^b^	Better to see patients in person for diagnosis	3

^a^ Total number of times the theme was mentioned across all interviews.

^b^ Overlap with Table 3—Limitations of telehealth.

CHM, Chinese herbal medicine.

#### Information sources

##### The use of a wide variety of information sources

Table 2 shows the variety of anecdotal sources that participants used in devising CHM formulas. Many reported that they obtained information from China through collegial networks—“I was most influenced by colleagues from China who I know personally and respect as people who are good in the classical symptoms of differential diagnosis” (PC 14), “but I want to say it was the end of January, I had a webinar with my primary herb teacher, who lives in Nanjing. And she gave us a lot of information about what the Wuhan doctors were doing at that time” (PC 6).

In the early stages of the COVID-19 pandemic information about the use of CHM was rapidly disseminated through formal sources such as United States continuing education providers (e.g., eLotus^27^) and the Lantern journal^28^—“it was amazing actually to have such senior practitioners, doctors, and professors sharing their insight and sharing a more nuanced pattern differentiation” (PC 3), “It was so valuable. I don't know if I would've been as successful and effective without that information” (PC 13). John Chen^29^ and Heiner Fruehauf^30^ are recognized CHM scholars who were involved in this dissemination—“Well, the first thing that was released, that I think that I read, was a brief by an herbalist called Heiner Fruehauf” (PC 3), “I saw the information coming out, so I attended like the John Chen lecture” (PC 1), “but what Dr. Chen did is he reviewed that for us, and then he added modern pharmacological information” (PC 10).

Usage of scientific studies was relatively low despite most participants being aware of their existence—“I didn't really pay attention to that, no” (PC 6), “Yeah, I did, and I ignored them (PC 1),” “I think I read them and I took them sort of under advisement, but it didn't really change how I diagnose and treat my patients” (PC 9). Participants that did use the studies found them useful for both clinical application and to understand pathomechanism—“Yes, I would read through those studies. My brother is a research scientist and we would talk about a lot of that” (PC 5), “I was reading all the Western studies and I was trying to understand the virus and I was trying to understand the pathomechanism to the virus. And I was trying to understand how does it move through the system and how does it correlate to what we know in the medicine?” (PC 1).

Many reasons were provided as to why the scientific studies were not used—“I would say at that point, not very much because I'd already been treating people and I was somewhat using my own—you know, I was basically using my own experience and how they were responding at that point” (PC 12).

They're not really organized in a way that makes sense to how I practice. Like because they're usually just like the question will be does this formula work on COVID, but the thing is that there's 15 different formulas that you might use on COVID based on how it's presenting, so I don't often find a lot of useful information in those studies unless they're doing a full differentiation (PC 3).

it was a very—only a minor impact on how I practice Chinese medicine. I think part of the question that you're kind of asking here is like how do I use or how do I incorporate evidence-based medicine in my practice of Chinese medicine. And for me, the evidence base that I rely on the most is the clinical case records of my teacher, my grandteacher, my greatgrandteacher, and the kind of like the core texts of Chinese medicine (PC 2).

#### Diagnostic and treatment decision-making

The various influences and approaches to diagnosis and determination of treatment approaches: Table 3 outlines the themes associated with the participant's diagnostic and treatment decision-making. Most participants reported that they used first principles meaning they relied upon a systematic approach to diagnosis that underlies EAM treatment of all conditions. This is often expressed as “treat what you see.” “I looked at their symptoms and their sort of—their Chinese medical pattern, and I went based on the best herbal formulas for each patient” (PC 5), “So, the way we made up our different treatment protocols were through examining the signs and symptoms that people were presenting,” “patients were my primary source of information” (PC 14).

I treated the patients with COVID the same way I treat all my patients for whatever they come in with. Basically it is collecting the landscape of signs and symptoms that are presenting, the diagnostics of tongue and pulse. With that I put together come up with a diagnosis. Then the treatment principle. And that leads me towards what formula (PC 4).

Participants expressed confidence in their own skills and success with CHM formulas that they had designed themselves—“Because I'm an experienced practitioner, and know how to write a prescription,” “I worked through like my own particular lens of viewing patients and the disease process that they were going through” (PC 2). Their treatment decisions were influenced by a variety of sources, including mentors and colleagues, articles, and the use of CHM in previous epidemics. When asked whether patient COVID test status influenced their treatment decisions the majority reported that it did not—“I treat what I see, so the positive test didn't really influence how I treated them” (PC 9).

#### Practitioner experience

The personal experiences of the participants treating patients during the pandemic: Participants reported on their experience treating patients during the COVID-19 pandemic (Table 4). Many reported a high degree of collaboration and a coming together of the global EAM community. Most used telehealth, and some expressed that seeing patients in-person was preferable. Treatment success was often reported as patients not being admitted to hospital or progressing into long-COVID—“Through the whole pandemic I was able to keep people out of the hospital” (PC 1), “I would say those people were on the cusp of being long haulers, and most of them got better very quickly with the herbs” (PC 12). Some participants treated patients who had received a positive COVID test, delivered CHM to patient's homes (due to disruption of delivery services—Table 4) —“I was doing a little herbal Door Dash around town” (PC 9), used CHM to prevent serious illness associated with possible COVID-19 infection, and worked within both an EAM and biomedical framework.

#### Resources and supplies

Barriers and limitations associated with resources and supplies: Most participants reported on challenges associated with various pandemic related barriers and limitations (Table 5). These included supply chain interruptions to CHM supplies and the resultant need to substitute herbs and stock up their own CHM pharmacies—“You know, from those early studies, if there were herbs or formulas that I thought that maybe I might need, I stocked up quite a bit” (PC 12). The use of telehealth was identified as a limitation—“Well, limitations were generally not being able to see people in person. Right, which means that you can't confirm pulse, necessarily, or see them as readily, or change the formulas as quickly, right, because you're mailing” (PC 3). Patient lack of access to COVID testing was also often experienced.

## Discussion

Their study suggests that licensed acupuncturists were treating COVID patients before the first official reported case in the United States. This is unsurprising because it has been estimated that as many as 80% of Americans that sought care for flu-like illnesses in March 2020 actually had COVID.^31^ Participants in their study covered all four U.S. geographic regions,^32^ and most started treating COVID patients before or during March 2020.

Before studies about the effectiveness and efficacy of CHM being published in the scientific literature, participants extensively accessed anecdotal information about the use of CHM for COVID-19 from the disseminated information within the United States and through *ad hoc* networks based on prior professional relationships with colleagues in China. Participants were aware of the scientific studies, and half of them used these despite recognition of their limitations in terms of relevance to their practice. Similar outcomes were seen from their survey undertaken with 103 licensed acupuncturists prescribing CHM for patients with symptoms likely related to COVID-19.^17^ In this study participants reported a wide variety of different sources being used to inform their CHM prescribing with information from colleagues, text books, continuing education courses, herbal medicine companies, and Chinese medicine journals being the most utilized. In addition, 50% of the participants used their own clinical experience.

A first principles approach to treatment was prevalent. Participants were confident in their own clinical skills and used the same approach to diagnosing and treating that they used with all conditions, what is often referred to in EAM as—treat what you see. The pandemic caused many limitations to effective treatment, with most being associated with the use of telehealth, getting CHM supplies, and delivering CHM to patients.

The preference for anecdotal information and reliance upon clinical experience in preference to the scientific studies can be understood in reference to several factors. As stated by the participants, the scientific studies were published after they had started treating patients and gaining confidence and experience with using CHM for COVID-19 treatment. Another issue that likely impacts this is the overall sentiment that licensed acupuncturists have toward the scientific research.^33,34^

Despite a growing evidence base to support the use of acupuncture^35,36^ and efforts by the National Institutes of Health who funded research programs within complementary and integrative health institutions aimed at increasing research literacy, there are still significant cultural barriers to embracing the use of evidence to inform clinical practice.^33,34,37^ Some of this relates to the barriers and limitations in applying evidence-based medicine to EAM^38–42^ due to the significantly different paradigms of biomedicine and EAM.^40,41^ EAM uses a whole person health^43–45^ approach in which illness is assessed in relation to an assessment of the whole physical, mental, and spiritual health of an individual. This contrasts with the more reductionist approach of biomedicine and science. Applying scientific models used for clinical research to assess the efficacy and effectiveness of EAM is problematic^41^ because these studies usually don't use EAM diagnostic approaches or tailor treatments to individual participants. Such issues weaken the external validity of the research and its ability to inform real world clinical practice.

In studies assessing the efficacy and effectiveness of CHM for COVID-19 these issues are especially problematic.^45^ CHM formulas are individualized to address specific patient symptoms and presentation.^46,47^ COVID-19 infection presents with a broad range of associated symptoms.^48^ The CHM formulas used in the COVID-19 trials were designed to treat common COVID-19 symptoms and were not individualized or modified over time. COVID-19 pathology is associated with a rapidly changing symptom picture. In real world clinical practice EAM practitioners would modify CHM formulas as the symptoms changed. In our study many participants talked about this and the importance of delivering modified formulas to patients in a timely manner—“I'd fill the formulas and hop in my car and drive it to their doorstep, which was great, ‘cause I could do it—I could change it every couple of days as their symptoms evolved” (PC 9).

Given the large amount of information that was disseminated within the EAM global community, the lack of scientific research in the early stages of the pandemic, and the significant challenges associated with using scientific research to inform patient care, it is not surprising that acupuncturists did not rely on the scientific studies. Their study shows that most of the participants knew of these studies but were not guided by them in designing CHM formulas to any significant extent. However, the scientific studies were often used to understand the pathology of the viral infection and to compare and validate their own treatment strategies.

This study has implications for evidence-based medicine as it is applied to ancient medical systems that existed well before modern medicine and science. Within the contemporary evidence-based medicine model^49^ and evidence pyramid,^50^ collective experience of the profession and observational research is deemed inferior to randomized controlled trials and systematic reviews. Within this paradigm the well documented theories and diagnostic and treatment methodologies of EAM collected over thousands of years of patient observation would likely not be considered a form of observational research. But is it not actually very similar to modern observational research approaches such as the case series?

During the COVID-19 pandemic this information along with the collective experience and knowledge of the EAM profession was initially the only information to base CHM clinical practice upon. This also encompassed the collective experience and knowledge of the profession gained from treating patients throughout prior epidemics over several millennia. Such knowledge has been instrumental to the development of EAM theories and is well documented in ancient EAM texts such as the *Huang Di Nei Jing*.^8^ More recently CHM was used in the 2003 SARS epidemic^7^ and the 2009 H1N1 influenza pandemic.^16^

An especially impressive and impactful aspect of their study was the benevolent coming together of the global EAM community and the open sharing of information during a time of strained intercountry relationships and finger pointing as to the origin of the virus. “it was just remarkable, the amount of, and just open free information sharing, like just totally benevolent information sharing on the part of like very, very like famous and in demand and busy doctors was incredible” (PC 3). What the authors saw within the global EAM community was a true alignment with the values expressed in all health care professional oaths. Given the mounting evidence of effectiveness of CHM for COVID-19^10–12^ perhaps a reevaluation of the value of traditional medicines and their theories and paradigms is warranted.

### Limitations

This study has several limitations. The interpretation of themes in the interview data may have been influenced by the investigators being licensed acupuncturists and CHM practitioners. Demographic data on the participants (age, years in practice, number of patients with symptoms likely related to COVID-19 that were treated) was not collected, and therefore, the participant sample may not accurately represent all U.S. licensed acupuncturists that were prescribing herbs for patients with symptoms likely related to COVID-19.

## Conclusions

CHM was likely used by licensed acupuncturists in the United States to treat patients with symptoms related to COVID-19 before the first official U.S. reported case. Various information sources were used along with practitioner experience and a direct approach of treating presenting signs and symptoms (“treat what I see”). The information sources informing their treatment strategies included anecdotal information from China disseminated through professional networks and EAM websites and journals, and to a lesser extent scientific studies evaluating the effectiveness of CHM for COVID-19. The latter were generally not deemed useful for informing patient care because treatment had been initiated before they were published and because of limitations associated with the research and the ability to apply it to real world practice. However, these studies were used to understand the pathomechanism of the virus.
